# 27-Hydroxycholesterol-induced EndMT acts *via* STAT3 signaling to promote breast cancer cell migration by altering the tumor microenvironment

**DOI:** 10.20892/j.issn.2095-3941.2019.0262

**Published:** 2020-02-15

**Authors:** Kailin Jiao, Jing Zhen, Maoxuan Wu, Mengying Teng, Keke Yang, Qian Zhou, Chunyan Hu, Ming Zhou, Yuan Li, Zhong Li

**Affiliations:** ^1^Department of Nutrition and Food Hygiene, School of Public Health, Nanjing Medical University, Nanjing 211166, China

**Keywords:** 27-Hydroxycholesterol, endothelial to mesenchymal transition, STAT3 acetylation, tumor microenvironment, breast cancer, migration

## Abstract

**Objective:** The endothelial to mesenchymal transition (EndMT) plays a major role in cancer metastasis by regulating the complexity of the tumor microenvironment (TME). Here, we investigated whether 27-hydroxycholesterol (27HC) induces EndMT in endothelial cells (ECs).

**Methods:** EndMT markers in the human microvascular endothelial cell-1 (HMEC-1) cell line and human umbilical vein endothelial cells (HUVECs) stimulated with 27HC were evaluated with Western blot. Epithelial to mesenchymal transition (EMT) markers in breast cancer (BC) cells cultured in conditioned medium were investigated with quantitative real time polymerase chain reaction (qRT-PCR). The MMP-2 and MMP-9 mRNA expression and activity were detected with qRT-PCR and gelatin zymography assays, respectively. The effect of activated STAT3 on 27HC-induced EndMT was validated by Western blot, immunofluorescence staining, and cell transfection assays. The migration ability of BC cells was evaluated with Transwell assays.

**Results:** We found that 27HC induced EndMT in HMEC-1 and HUVECs, and 27HC-induced EndMT facilitated EMT and BC cell migration. The 27HC-induced EMT of BC cells also promoted EndMT and HUVEC migration. Investigation of the underlying molecular mechanisms revealed that STAT3 knockdown repressed EndMT in HUVECs as well as migration in BC cells induced with 27HC. In addition, C646 and resveratrol, inhibitors of STAT3 acetylation, repressed the expression of Ac-STAT3, p-STAT3, and EndMT markers in HUVECs exposed to 27HC; these HUVECs in turn attenuated the migration ability of BC cells in 27HC-induced EndMT.

**Conclusions:** Cross-talk between 27HC-induced EndMT and EMT was observed in the TME. Moreover, activation of STAT3 signaling was found to be involved in 27HC-induced EndMT.

## Introduction

The tumor microenvironment (TME) consists of extra-cellular matrix (ECM) components, cellular components such as fibroblasts, immune cells and endothelial cells (ECs), nerves, and vessels^1^. The TME plays a crucial role in tumorigenesis, tumor development, and therapeutic resistance^2^. The interaction between tumor cells and their microenvironment has been studied extensively. The vasculature within the TME is crucial for tumor formation and progression *via* endothelial dysfunction^3^. Recent studies have indicated that dysfunctional ECs upregulate vascular endothelial growth factor (VEGF) and syndecan-1 in the tumor stroma, thereby promoting angiogenesis and stimulating migration, epithelial to mesenchymal transition (EMT), and cholangiocarcinoma cell invasion^4,5^.

Endothelial to mesenchymal transition (EndMT), a process similar to EMT, is defined as a loss of endothelial specification and gain of mesenchymal characteristics^6^. During EndMT, cells change from an endothelial cell morphology to a spindle-like shape^7^. The expression of the endothelial markers VE-cadherin and CD-31 decreases, and mesenchymal markers such as vimentin, alpha-smooth muscle actin (α-SMA), fibroblast-specific protein 1 (FSP-1), and matrix metalloproteases (MMPs) are upregulated^8^. The polarity and adhesion of ECs decrease in the process of EndMT. Damage to endothelial junctions causes migration of ECs towards the surrounding tissues^9^. EndMT is involved in complex fibrotic disorders, such as systemic sclerosis, cardiac fibrosis, kidney fibrosis, pulmonary fibrosis, and liver and portal vein fibrosis^10^. EndMT is also crucial for the development of various cancers. As much as 40% of cancer-associated fibroblasts (CAFs) are produced during EndMT^11^. In addition, CAFs secrete inflammatory cytokines that indirectly regulate the migration and invasion of cancer cells^12^.

Epidemiological studies have suggested that dietary cholesterol accumulation promotes the development of breast cancer (BC)^13^. Extensive research has indicated that 27-hydroxycholesterol (27HC), an abundant primary metabolite of cholesterol, facilitates the proliferation of estrogen receptor-positive BC cells and metastasis of BC^14–17^. In a previous study from our group, we showed that 27HC promotes the migration and invasion of BC cells by inducing signal transducer and activator of transcription 3 (STAT3) phosphorylation-induced EMT^18^. However, whether 27HC can induce EndMT in ECs and affect the TME remains unclear. The underlying regulatory mechanisms of EndMT remain undefined. Studies have suggested that Notch, β-catenin, and Wnt signaling are involved in EndMT. The transforming growth factor β (TGF-β) signaling pathway is also involved in EndMT^8^. A recent study has shown that interleukin-6 (IL-6)-mediated STAT3 phosphorylation plays a role in EndMT, thus inducing cancer growth^19^.

Here, we investigated whether 27HC might induce EndMT in ECs and whether STAT3 acetylation might be involved in the process of EndMT. Anti-EndMT may serve as a potential therapeutic strategy to inhibit BC metastasis caused by 27HC.

## Materials and methods

### Cell culture and reagents

The human microvascular endothelial cell-1 (HMEC-1) cell line and human umbilical vein endothelial cells (HUVECs) were obtained from the Institute of Biochemistry and Cell Biology, Shanghai Institutes for Biological Sciences. The human BC cell lines MCF7, T47D, and MDA-MB-231 were purchased from the American Type Culture Collection (ATCC, Rockville, MD, USA). HMEC-1 cells and HUVECs were cultured in ECM medium (Invitrogen, Carlsbad, CA, USA); MCF7 and T47D cells were cultured in Dulbecco’s modified Eagle’s medium/Ham’s F-12 nutrient mixture (1:1 mixture, Life Technologies/Gibco, Grand Island, NY, USA), and MDA-MB-231 cells were cultured in L15 medium (Gibco). The media were supplemented with 10% fetal bovine serum (FBS, Sigma), 100 units/mL penicillin (Beyotime Co. Ltd.), 100 mg/mL streptomycin (Beyotime Co. Ltd.) and 30 mg/mL endothelial cell growth supplement (for HMEC-1 cells and HUVECs, Sigma-Aldrich, St. Louis, MO, USA). HMEC-1, HUVEC, MCF7, and T47D cells were grown in a humidified incubator containing 5% CO_2_ at 37 °C, and MDA-MB-231 cells were cultured in a humidified incubator containing no CO_2_ at 37 °C. The 27HC (purity ≥ 99.9%) was purchased from Santa Cruz Biotechnology (Santa Cruz, CA, USA) and dissolved in absolute ethanol to a stock concentration of 20 mM, then stored at −80 °C. TGF-β1 was purchased from Peprotech (Rocky Hill, NJ, USA) and diluted in citric acid (10 mM, pH = 3.0) to a stock concentration of 20 µg/mL, then stored at −80 °C. C646 was purchased from MedChem Express (USA) and diluted in dimethyl sulfoxide to a stock concentration of 10 mM, then stored at −80 °C. Resveratrol (purity = 98%) was purchased from Xuhuang Biotechnology Co. Ltd. (China) and dissolved in absolute ethanol to a stock concentration of 20 mM, then stored at −80 °C.

### Cell viability assays

HMEC-1 cells and HUVECs (1 × 10^6^ cells) were cultured in 96-well plates for 24 h, then treated with 0, 0.5, 1, 2, 5, 10, or 15 µM 27HC for 48 h. Next, the culture solution containing 27HC was replaced with 100 µL of cell culture medium containing 10% Cell Counting Kit-8 reagent (Beyotime Co. Ltd.) for 1 h. The absorbance at 450 nm was examined with a multi-well plate reader (Model 680; Bio-Rad; Hercules, CA, USA).

### Detection of cell morphology

HMEC-1 cells and HUVECs (1 × 10^6^ cells) were cultured in cell dishes for 24 h and treated with 0, 2.5, 5 µM 27HC or 10 ng/mL TGF-β1, for 48 h or for 5 passages. The morphology of ECs was determined with an inverted microscope (Olympus, Tokyo, Japan).

### Western blot

Radioimmunoprecipitation assay buffer (Beyotime Co. Ltd.) was used to extract the total protein. A Bicinchoninic Acid Kit (Beyotime Co. Ltd.) was used to estimate the protein concentrations. Proteins (20 µg) were separated by 10% sodium dodecyl sulfate-polyacrylamide gel electrophoresis (SDS-PAGE) and transferred to polyvinylidene fluoride membranes (Millipore, Billerica, MA, USA). After being blocked with 5% non-fat milk, the membranes were incubated with the primary antibodies (**Supplementary Table S1**) overnight at 4 °C, followed by horseradish peroxidase-conjugated secondary antibodies (Yifeixue Bio Tech, Nanjing, China; 1:5000) for 1 h at 37 °C. Immune complexes were detected by enhanced chemiluminescence.

### Gelatin zymography

Treated HUVECs were cultured in ECM medium containing 1% FBS and maintained in a humidified incubator containing 5% CO_2_ at 37 °C. Then, the culture supernatants were collected and centrifuged. Proteins in the conditioned medium were separated with SDS-PAGE (10%) in a resolving gel containing 1 mg/mL gelatin (Sigma-Aldrich) under non-reducing conditions. The gels were washed 3 times in 2.5% Triton X-100/50 mM Tris–HCl, then subjected to electrophoresis (pH 7.6) and incubation in renaturation solution containing 0.15 M NaCl/10 mM CaCl_2_/50 mM Tris–HCl, and 0.05% NaN_3_ (pH 7.6) in an incubator without CO_2_ for 1 h at 37 °C. Gels were then stained with 0.005% Coomassie Blue R250 for 3 h and destained with 10% acetic acid and 10% isopropanol. MMP-2 and MMP-9 bands were detected on the slab gel^18^.

## Quantitative real-time polymerase chain reaction (qRT-PCR)

TRIzol (Yifeixue Bio Tech) reagent was used to extract total RNA from cells. Primers (**Supplementary Table S2**) were synthesized by Tsingke (Nanjing, China). Total RNA (2 µg) was reverse transcribed into cDNA with AMV Reverse Transcriptase (ABM, Canada) according to the following procedure: 25 °C for 10 min, 42 °C for 15 min, and 85 °C for 5 min. Synthesis of first-strand cDNA was carried out with SYBR Green Master Mix (Vazyme Biotech Co, Ltd.). qRT-PCR assays were performed with Light Cycler 96 SYBR Green I Master Mix (Roche) for 35 cycles at 95 °C for 300 s, 95 °C for 10 s, and 60 °C for 30 s.

### Transwell assays

Cell migration was assessed with Transwell chambers (8 µm pore size, Becton Dickinson, Franklin Lakes, NJ, USA). The conditioned medium samples were placed in the lower chambers with 15% FBS. Treated cells were seeded into the upper chambers in culture medium with 2% FBS. HUVECs were incubated in 5% CO_2_ at 37 °C for 24 h, and T47D cells were incubated in 5% CO_2_ at 37 °C for 48 h or 72 h. MDA-MB-231 cells were incubated without CO_2_ for 24 h at 37 °C. The upper surface cells in the chamber were removed, and migrating cells were counted with Stat Monitor in Photoshop in 5 random fields.

### Immunofluorescence staining assays

Treated cells were fixed in 4% paraformaldehyde for 10 min and permeabilized with 0.2% Triton X-100 for 10 min at room temperature. Then, cells were incubated with primary antibodies overnight at 4 °C. Cells were subsequently incubated with Alexa Fluor-conjugated secondary antibodies (Beyotime Co. Ltd.) for 2 h at 37 °C, and nuclei were stained with Hoechst 33342 (Beyotime Co. Ltd.). Cell fluorescence was examined under a fluorescence microscope (Olympus).

### Cell transfection assays

The siRNAs were purchased from Santa Cruz Biotechnology (**Supplementary Table S3**). HUVECs were seeded in cell culture dishes (5 × 10^5^ cells) for 24 h and transiently transfected with a mixture of Lipofectamine 2000 (Invitrogen) and si-Con (negative control siRNA) or si-STAT3 for 6 h. The medium was removed, and transfected cells were incubated for another 48 h in fresh medium containing 10% FBS. Cells were used for subsequent experiments.

### Statistical analysis

GraphPad-6.0 (GraphPad Software, Inc., La Jolla, CA, USA) was used to compare data sets. Values were presented as the mean ± standard deviation (SD). The variance among groups was analyzed with a two-tailed Student’s *t*-test or one-way analysis of variance (ANOVA), and *P* values < 0.05 were considered statistically significant.

## Results

### EndMT is induced in ECs by short- and long-term treatment with 27HC

**Figure 1:**
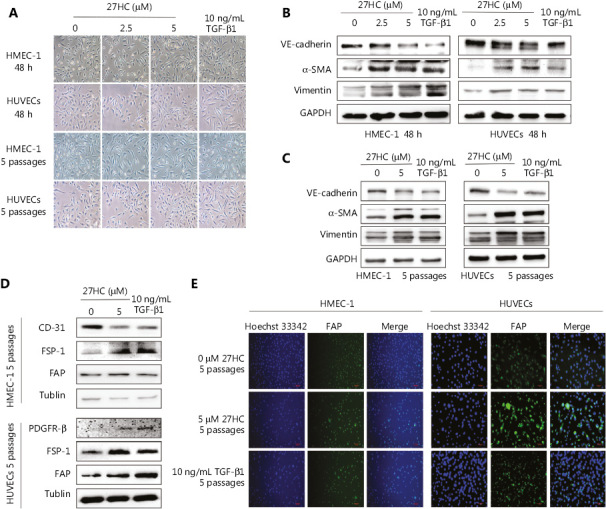
Endothelial to mesenchymal transition (EndMT) is induced in endothelial cells (ECs) by short- and long-term treatment with 27-hydroxycholesterol (27HC). (A) HMEC-1 and human umbilical vein endothelial cells (HUVECs) were exposed to 0, 2.5, or 5 μM 27HC for 48 h or 5 passages. Compared with untreated cells, ECs treated with 27HC had a fibroblast-like phenotype. Transforming growth factor beta 1 (TGF-β1) (10 ng/mL) treatment was used as a positive control. (B) HMEC-1 cells and HUVECs were exposed to 0, 2.5, or 5 μM of 27-hydroxycholesterol (27HC) for 48 h. Western blot analyses of VE-cadherin, vimentin, and α-SMA in ECs. Transforming growth factor beta 1 (TGF-β1) (10 ng/mL) treatment was used as a positive control. (C) HMEC-1 cells and HUVECs were exposed to 0 or 5 μM of 27HC for 5 passages. The expression of VE-cadherin, vimentin, and α-SMA was determined by Western blot. TGF-β1 (10 ng/mL) was used as a positive control. (D) HMEC-1 cells were exposed to 0 or 5 μM 27HC for 5 passages. Western blot analyses of protein expression of CD-31, FSP-1, and FAP in HMEC-1. TGF-β1 (10 ng/mL) treatment was used as a positive control. HUVECs were exposed to 0 or 5 μM of 27HC for 5 passages. The expression of PDGFR-β, FSP-1, and FAP was determined by Western blot. TGF-β1 (10 ng/mL) was used as a positive control. (E) HMEC-1 cells and HUVECs were treated with 0 or 5 μM 27HC for 5 passages. Immunofluorescence staining analyses of the expression of FAP in ECs. TGF-β1 (10 ng/mL) treatment was used as a positive control.

We initially explored the effects of 27HC on the proliferation of HMEC-1 cells and HUVECs, and observed that 27HC did not significantly suppress EC cell viability at 0–5.0 µM for 48 h, as shown in **Supplementary Figure S1**. We chose 5 µM as the maximum 27HC concentration for further experiments. To explore the potential role of 27HC in EndMT in ECs, we subjected HMEC-1 cells and HUVECs to short-term (48 h) and long-term (5 passages) 27HC treatments, respectively. Under the 27HC conditions, the phenotype of ECs gradually changed from an endothelial morphology to elongated spindle-shaped structures. TGF-β1 treatment was used as a positive control (**Figure 1A**). Detection of proteins associated with EndMT revealed that the endothelial marker VE-cadherin was downregulated, whereas the mesenchymal markers vimentin and α-SMA were upregulated in the 27HC groups; the TGF-β1 group was used as a positive control (**Figure 1B, 2C**). Collectively, these results indicated that 27HC induced the process of EndMT in vascular ECs.

EndMT is an important source of CAFs^11^. We determined the CAF markers in HMEC-1 cells and HUVECs at 0 or 5 µM 27 HC for 5 passages, using the TGF-β1 group as a positive control. As shown in **Figure 1D**, in the 27HC treated group compared with the untreated group, CD-31 (a negative marker of CAFs) was diminished, whereas FSP-1 and fibroblast activation protein (FAP) (positive markers of CAFs) were elevated in HMEC-1 cells. In addition, platelet derived growth factor receptor beta (PDGFR-β), FSP-1, and FAP—positive markers of CAFs—were higher in HUVECs treated with 27HC than in the untreated cells. As shown in **Figure 1E**, immunofluorescence staining assays revealed that FAP was enhanced in HMEC-1 cells and HUVECs exposed to 27HC compared with the untreated cells. Next, we detected the mRNA expression of CAFs in HMEC-1 cells and HUVECs at 0 or 5 µM 27HC for 5 passages. As shown in **Supplementary Figure S2**, the expression of IL-6 and fibroblast growth factor (FGF-2) was higher in HMEC-1 cells and HUVECs treated with 27HC than in untreated cells, and TGF-β expression increased in HMEC-1 cells, as promoted by 27HC.

### 27HC-induced EndMT facilitates EMT and the migratory capacity of BC cells

**Figure 2:**
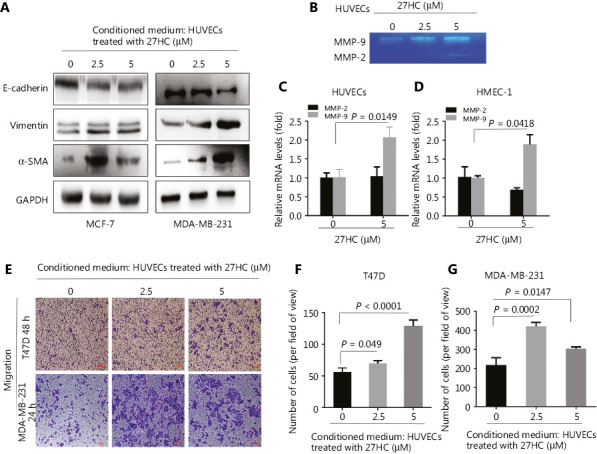
27-Hydroxycholesterol (27HC)-induced endothelial to mesenchymal transition (EndMT) facilitates epithelial to mesenchymal transition (EMT) and increases the migratory capacity of breast cancer (BC) cells. (A) MCF-7 and MDA-MB-231 cells were cultured in conditioned medium collected from human umbilical vein endothelial cells (HUVECs) treated with 27HC (0, 2.5, or 5 μM). Western blot analyses of E-cadherin, vimentin, and α-SMA in BC cells. (B) MMP-9 and MMP-2 levels in HUVECs stimulated with 27HC (0, 2.5, or 5 μM) were investigated with gelatin zymography assays. (C, D) Quantitative real time polymerase chain reaction (qRT-PCR) analyses in triplicate of MMP-9 and MMP-2 mRNAs in HMEC-1 cells and HUVECs exposed to 27HC (0 or 5 μM). (E) HUVECs were treated with 0, 2.5, or 5 μM of 27HC for 48 h, and then conditioned medium was collected (without 27HC). T47D and MDA-MB-231 cells were exposed to the conditioned medium for 48 h and 24 h, respectively. Transwell assays of BC cells were performed. Scale bars = 100 μm. (F, G) The numbers of T47D and MDA-MB-231 migratory cells were determined.

Our previous study has indicated that 27HC induces the invasion and migration of BC cells by increasing MMP-9 expression and promoting EMT^18^. To explore whether EMT in BC cells might be promoted by 27HC-induced EndMT, we cultured MCF-7 and MDA-MB-231 cells in conditioned medium collected from 27HC-treated HUVECs. The expression of the epithelial marker E-cadherin decreased, whereas that of the mesenchymal markers vimentin and α-SMA increased (**Figure 2A**). Next, we determined the mRNA expression and activity of MMP-9 and MMP-2 in ECs treated with 27HC. Gelatin zymography indicated that the activity of MMP-9 increased in HUVECs exposed to 27HC, and the mRNA level of MMP-9 increased in HMEC-1 cells and HUVECs treated with 27HC (*P* = 0.0418, *P* = 0.0149). However, no obvious differences in MMP-2 were observed (**Figure 2B–2D**). Furthermore, the conditioned medium collected from 27HC-treated HUVECs was added to T47D (48 h) and MDA-MB-231 cells (24 h), and the migration ability of both BC cell lines consequently increased (**Figure 2E–2G**). These findings demonstrated that 27HC-induced EndMT promotes the migration of BC cells by inducing EMT and activating MMP-9.

**Figure 3:**
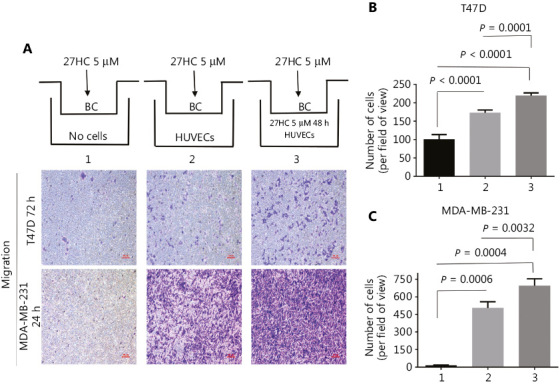
The effect of human umbilical vein endothelial cells (HUVECs) on migration of breast cancer (BC) cells. In the co-culturing model, extra-cellular matrix (ECM) medium with 15% fetal bovine serum (FBS) (1. No cells; 2. Containing 1 × 10^4^ HUVECs; 3. Containing 1 × 10^4^ HUVECs exposed to 5 μM 27-hydroxycholesterol (27HC)). HUVECs were placed at the bottom of the wells in a 24-well plate, and then T47D and MDA-MB-231 cells treated with 5 μM 27HC were seeded on the upper side of the membrane of the insert in culture medium with 2% FBS for 72 h and 24 h, respectively. (A) Cell migration determined by Transwell assays. Scale bars = 100 μm. BC: breast cancer cells. (B, C) Numbers of T47D and MDA-MB-231 migratory cells were evaluated.

To further explore the effect of HUVECs on BC cell migration, we used a co-culturing model (**Figure 3A**). In the HUVEC group, compared with the no HUVEC group, under treatment with 27HC, the number of T47D and MDA-MB-231 migratory cells significantly increased (*P* < 0.0001, *P* = 0.0006); furthermore, compared with the HUVEC group, HUVECs treated with 27HC showed significantly greater migratory ability of T47D and MDA-MB-231 (*P* = 0.0001, *P* = 0.0032) (**Figure 3A–3C**).

### 27HC-induced EMT of BC cells promotes EndMT and migration in HUVECs

**Figure 4:**
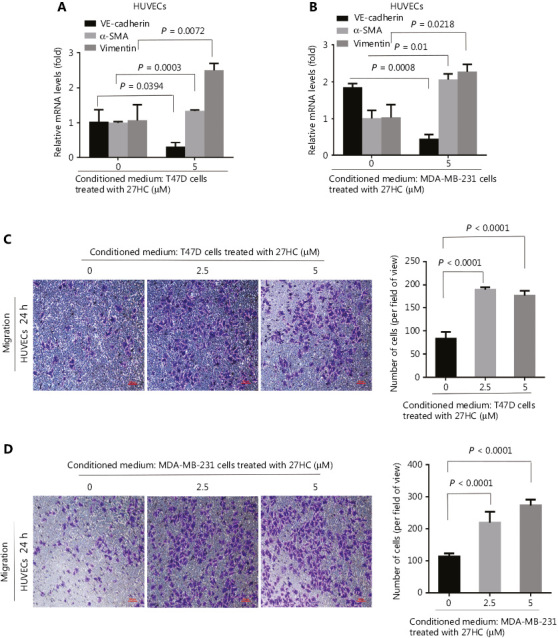
27HC-induced epithelial to mesenchymal transition (EMT) of breast cancer (BC) cells promotes endothelial to mesenchymal transition (EndMT) and migration in human umbilical vein endothelial cells (HUVECs). T47D and MDA-MB-231 cells were exposed to 0 or 5 μM 27HC for 48 h, and conditioned medium was collected. HUVECs were exposed to the conditioned medium for 24 h. (A, B) Relative mRNA expression of the endothelial marker VE-cadherin and the mesenchymal markers vimentin and α-SMA were assessed with quantitative real time polymerase chain reaction (qRT-PCR). (C, D) Transwell assays were performed, and numbers of migratory HUVECs were analyzed. Scale bars = 100 μm.

EMT and EndMT are critical factors that affect the TME and support cancer progression^20^. We hypothesized that 27HC might induce an amplification cascade in the TME. To confirm the effect of 27HC-induced EMT of BC cells on ECs, we cultured HUVECs in conditioned medium collected from 27HC-treated T47D and MDA-MB-231 cells. In the 27HC group compared with the untreated group, VE-cadherin mRNA expression was lower, whereas vimentin and α-SMA mRNA levels were higher (**Figure 4A, 4B**). The migration ability of HUVECs incubated (24 h) in conditioned medium collected from 27HC-treated T47D cells significantly increased (*P* < 0.0001) (**Figure 4C**). Similar effects were observed in response to conditioned medium collected from 27HC-treated MDA-MB-231 cells (*P* < 0.0001) (**Figure 4D**). Collectively, these results and our previous work suggest that 27HC-induced EMT of BC cells affects the TME by promoting EndMT and migration in vascular ECs.

### Activation of STAT3 signaling is involved in 27HC-induced EndMT

**Figure 5:**
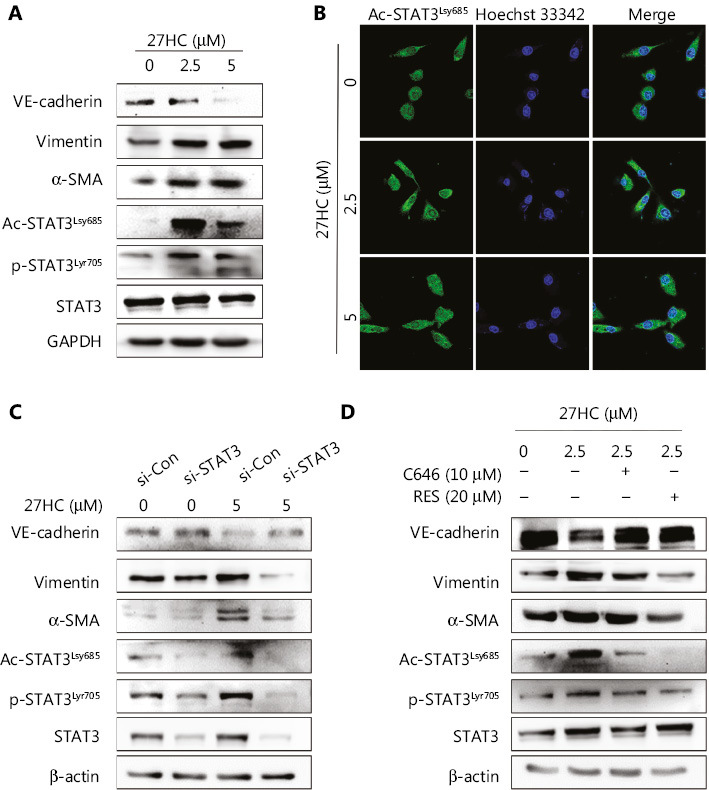
STAT3 acetylation and phosphorylation are involved in 27-hydroxycholesterol (27HC)-induced endothelial to mesenchymal transition (EndMT). Human umbilical vein endothelial cells (HUVECs) were treated with 0, 2.5, or 5 μM 27HC for 48 h. (A) The protein levels of Ac-STAT3^Lsy685^, p-STAT3^Tyr705^, and EndMT markers (VE-cadherin, vimentin, and α-SMA) were determined by Western blot analysis. (B) Immunofluorescence staining of Ac-STAT3^Lsy685^ (green) expression. (C) HUVECs were transiently transfected with 25 nM of si-Con or si-STAT3 for 6 h, then exposed to 0 or 5 μM 27HC for 48 h. The protein levels of Ac-STAT3^Lsy685^, p-STAT3^Tyr705^, STAT3, and EndMT markers (VE-cadherin, vimentin, and α-SMA) were examined by Western blot. (D) HUVECs were pre-treated with C646 (0, 10 μM) and RES (0, 20 μM) for 3 h, then exposed to 0 or 2.5 μM 27HC for another 48 h. HUVECs were then harvested, and proteins were examined by Western blot with the indicated antibodies.

A previous study has indicated that HSPB1-dependent JAK-STAT signaling may play a role in radiation-induced EndMT, as suggested by increased STAT3 phosphorylation associated with radiation-induced EndMT^21^. Therefore, we examined the expression of Ac-STAT3 and p-STAT3 in 27HC-induced EndMT. As shown in **Figure 5A**, the protein levels of Ac-STAT3^Lsy685^ and p-STAT3^Tyr705^ were markedly higher in 27HC-treated HUVECs. STAT3 modulates histone acetylation by recruiting histone acetyltransferase to a particular gene promoter, thereby modulating gene transcription^22,23^. Immunofluorescence assays revealed that enhanced Ac-STAT3 showed marked translocation into the nucleus in HUVECs treated with 27HC (**Figure 5B**).

To further confirm that activated STAT3 was responsible for 27HC-induced EndMT, we knocked out STAT3 by using three different si-RNAs specific to STAT3 in HUVECs (**Supplementary Figure S3**) and chose si-RNA 1 for use in subsequent experiments. Compared with si-Con transfected cells, cells with STAT3 knockdown showed attenuation of the 27HC-induced expression of α-SMA and vimentin, and decreased expression of VE-cadherin (**Figure 5C**). We additionally treated cells with C646, a selective inhibitor of p300 (histone acetyltransferase), which represses p300-mediated STAT3 acetylation^24^, and with resveratrol (RES), a naturally occurring phytoalexin that inhibits STAT3 acetylation^25^. We found that STAT3 acetylation decreased in both the C646 and RES groups, and 27HC-induced EndMT was reversed. Moreover, the levels of p-STAT3 were regulated by Ac-STAT3 (**Figure 5D**). Together, these results indicated that STAT3 activation promotes EndMT in vascular ECs exposed to 27HC, and STAT3 acetylation might regulate phosphorylation.

### Inhibition of STAT3 signaling attenuates the effect of 27HC-induced EndMT on the migration of BC cells

**Figure 6:**
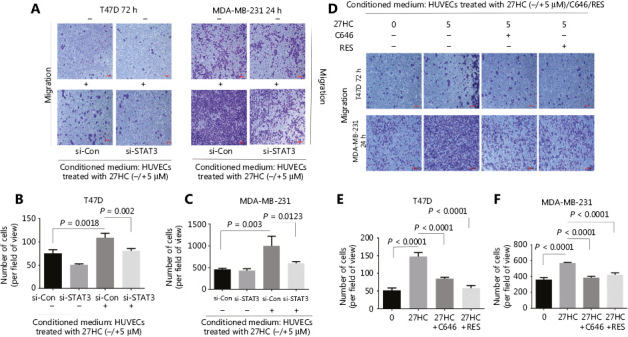
Inhibition of STAT3 signaling attenuates the effect of 27HC-induced endothelial to mesenchymal transition (EndMT) on the migration of breast cancer (BC) cells. Human umbilical vein endothelial cells (HUVECs) were transiently transfected with 25 nM si-Con or si-STAT3 for 6 h, then exposed to 0 or 5 μM 27-hydroxycholesterol (27HC) for another 48 h. Conditioned medium was collected (without 27HC). T47D and MDA-MB-231 cells were cultured in the conditioned medium for 72 h and 24 h, respectively. (A) Cell migration determined by Transwell assays. Scale bars = 100 μm. (B, C) Numbers of T47D and MDA-MB-231 migratory cells were evaluated. HUVECs were then pre-treated with C646 (0, 10 μM) and RES (0, 20 μM) for 3 h, and subsequently treated with 0 or 5 μM 27HC for another 48 h. Conditioned medium was collected. T47D and MDA-MB-231 cells were cultured in the conditioned medium for 72 h and 24 h, respectively. (D) Migration was evaluated with Transwell assays. Scale bars = 100 μm. (E, F) Numbers of T47D and MDA-MB-231 migratory cells were determined.

To further examine the effect of STAT3 signaling associated with 27HC-induced EndMT on the migration of BC cells, we knocked out STAT3 and treated HUVECs with 27HC. We then incubated T47D cells (72 h), and MDA-MB-231 cells (24 h) in conditioned medium collected from treated HUVECs, and assessed the migration ability of BC cells with Transwell assays. Dramatic attenuation of migration was observed for T47D cells treated with conditioned medium collected from STAT-3 knockout in HUVECs exposed to 27HC, compared with si-Con transfected HUVECs exposed to 27HC (*P* = 0.002) (**Figure 6A, B**); furthermore, similar results were observed in MDA-MB-231 cells (*P* = 0.0123) (**Figure 6A, C**).

Next, we incubated T47D cells (72 h) and MDA-MB-231 cells (24 h) in the conditioned medium collected from HUVECs treated with 0, 27HC, 27HC + C646 and 27HC + RES. In the 27HC + C646 and 27HC + RES groups compared with the 27HC group, a significant decrease was observed in the number of migratory T47D cells (72 h) and MDA-MB-231 cells (24 h) (*P* < 0.0001) (**Figure 6D–F**).

We also explored the effect of STAT3 on MMP9 in HUVECs with STAT3 knockdown and C646 or RES treatment. Knockdown of STAT3 decreased the level of MMP9 in HUVECs, and C646 or RES inhibited the level of MMP9 promoted by 27HC (**Supplementary Figure S4**). Together, these results indicate that STAT3 signaling is a key regulator of 27HC-induced EndMT, which in turn regulates the migration of BC cells.

## Discussion

Increasing evidence supports the role of TME alterations in BC progression^26^. EndMT plays an important role in the formation of the complex tumor stroma. TGF-β induces EndMT, a process in which the morphology of ECs changes to a spindle-like shape. This process is also involved in the regulation of EndMT markers^27^. In the present study, we investigated the effects of short and long exposure of ECs to 27HC. In agreement with the findings of TGF-β-induced EndMT, our results demonstrated that 27HC also induced EndMT. Loss of VE-cadherin during EndMT can suppress adherens junctions and increase permeability in ECs^28^. Tip cells migrate toward the surrounding tissue and form extensive vascular networks that facilitate tumor growth and metastasis^3^. CAFs are often characterized by the expression of α-SMA, and they secrete chemokines, tumor growth factors, and abundant ECM components, thus supporting BC progression^29,30^. In this study, we also observed that CAF markers were upregulated by 27HC in ECs. Therefore, EndMT may serve as a novel therapeutic target for BC progression promoted by 27HC.

A previous study has suggested that integrin-linked kinase overexpression downregulates MMP-9 during TGF-β2-induced EndMT, and elevated MMP-9 increases the invasive capacity of cancer cells^20^. Another study has shown that MMP-2 plays a major role in enhancing the motility of HMEC-1 cells during EndMT, although the expression and secretion of MMP-9 do not increase^31^. The results from the present study indicated that the mRNA expression and activity of MMP-9 were increased by 27HC-induced EndMT, and MMP-9 promoted the migration of BC cells; however, no changes in MMP-2 were observed. The factors responsible for the difference between MMP-9 and MMP-2 during 27HC-induced EndMT remain unknown, and further research is necessary. The complex crosstalk of the TME is involved in cancer progression. Therefore, we generated a co-culture model *in vitro*. Our findings showed that 27HC-induced EndMT promoted EMT and increased the migration of BC cells. HUVECs treated with 27HC significantly promoted BC cell migration. In addition, 27HC-induced EMT promoted EndMT and increased the migration of ECs. Collectively, these results indicate that 27HC induces TME alterations, thereby promoting BC cell migration.

The mechanisms underlying the role of EndMT are not fully understood. A recent study has shown that neural adhesion molecule L1 promotes EC proliferation and migration through the IL-6/JAK/STAT axis, and IL-6-mediated STAT3 phosphorylation is associated with EndMT and promotes cancer growth^19^. In a previous study, we demonstrated that 27HC induces EMT by promoting STAT3 phosphorylation and increases the migration and invasion of BC cells^18^. In the present study, Ac-STAT3 and p-STAT3 levels were higher in the 27HC group than in the control group, and knockdown of STAT3 attenuated 27HC-induced EndMT. These results suggested that 27HC activates STAT3 signaling and thereby regulates EndMT. Acetyltransferases and histone deacetylases can modulate STAT3 acetylation, and acetylated STAT3 can translocate from the cytoplasm to the nucleus, thus promoting the transcription of STAT3 target genes^32^. The present results indicated that 27HC stimulates the nuclear translocation of acetylated STAT3, as shown by immunofluorescence imaging (**Figure 5B**). Acetylated STAT3 may directly regulate STAT3 phosphorylation through the fasting-activated longevity protein sirtuin 1 (sirt1); moreover, sirt1 specifically deacetylates STAT3 and decreases STAT3 phosphorylation *in vitro* and *in vivo*^33^. Knockdown of p300 decreases STAT3 acetylation, and C646, a selective inhibitor of p300, inhibits p300-dependent STAT3 acetylation^24^. The phytoalexin RES has anti-inflammatory and chemopreventive effects, and it functions as a cyclooxygenase inhibitor; it can inhibit src tyrosine kinase and affect STAT3 activation in tumor cells. RES is therefore considered a potential therapeutic approach to decreasing STAT3 acetylation^25,34^. In this study, we evaluated the effects of C646 and RES on 27HC-induced EndMT through the suppression of STAT3 acetylation, and we found that STAT3 phosphorylation and EndMT were inhibited. In brief, STAT3 acetylation may play a major role in regulating 27HC-induced EndMT; however, the mechanisms regulating the association between STAT3 acetylation and STAT3 phosphorylation in 27HC-induced EndMT requires further investigation.

Activated STAT3 signaling plays an important role in the invasion and metastasis of tumors, and phosphorylated STAT3 affects cell adhesion ability, invasion, and metastasis in BC. MMP-9 expression is closely associated with tyrosine phosphorylated Stat3 in BC, and MMP-9 can bind CD44 and degrade the ECM, thus promoting metastasis^35,36^. STAT3 overexpression upregulates the EMT marker E-cadherin, whereas STAT3 knockdown significantly reverses the IL-6-mediated EMT phenotype of tumors^37,38^. Although STAT3 phosphorylation has been investigated extensively, the role of STAT3 acetylation in tumor progression has rarely been reported^39^. In the present study, we cultured BC cells with EC culture supernatants to indirectly study the effect of STAT3 signaling on cell migration induced by 27HC. Knockdown of STAT3 inhibited the BC migration promoted by 27HC-induced EndMT; moreover, suppression of STAT3 acetylation with C646 or RES inhibited cell migration. Therefore, STAT3 signaling-mediated EndMT promoted the migration of BC cells. Thus, STAT3 acetylation might be a major regulatory mode in physiological processes.

## Conclusions

**Figure 7:**
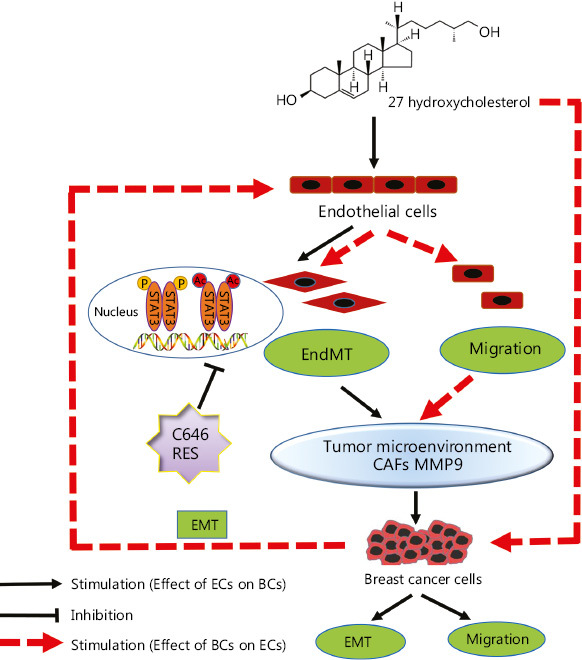
Schematic model of 27HC-induced endothelial to mesenchymal transition (EndMT). 27-Hydroxycholesterol (27HC) induced EndMT in vascular endothelial cells (ECs), and the process promoting epithelial to mesenchymal transition (EMT) and migration in breast cancer (BC) cells through alteration of the tumor microenvironment. In BC, 27HC-induced EMT promotes the EndMT and migration in ECs; moreover, 27HC activates the phosphorylation and acetylation of STAT3, thereby regulating EndMT of ECs, and C646 or RES inhibited the effect of 27HC.

The present study demonstrated that 27HC can induce EndMT and upregulate MMP-9, thereby promoting the migration of BC cells. Activation of STAT3 mediated 27HC-induced EndMT, as well as STAT3 acetylation, was found to play an important role by regulating STAT3 phosphorylation. However, further studies are needed to elucidate the regulatory relationship between Ac-STAT3 and p-STAT3 in 27HC-induced EndMT. We found that the interaction between 27HC-induced EndMT and EMT is involved in the migration of BC cells. C646 and RES can reverse EndMT and inhibit the migration of BC cells. A schematic model of 27HC-induced EndMT is shown in **Figure 7**. The present findings suggest that anti-EndMT may be an additional therapeutic strategy to combat BC progression promoted by 27HC, and STAT3 acetylation may be a novel regulatory mode for EndMT.
