# Supplementary material for: 27-Hydroxycholesterol-induced EndMT acts via STAT3 signaling to promote breast cancer cell migration by altering the tumor microenvironment
Source: Cancer Biol Med. 2020 Feb 15;17(1):88–100. doi: 10.20892/j.issn.2095-3941.2019.0262 (PMC7142833; doi:10.20892/j.issn.2095-3941.2019.0262)
Supplement: Supplementary file 1 [file cbm-17-088-s001.pdf]

Supplementary materials

Table S1 Antibodies used in this study

Names	Web Link	Source	Used
VE-cadherin antibody	https://www.cellsignal.com/products/primary-antibodies/ve-cadherin-d87f2-xp-rabbit-mab/2500?site-search-type=Products&N=4294956287&Ntt=ve-cadherin+antibody&fromPage=plp	Cell Signaling Technology	1:1000
α-Smooth muscle actin antibody	https://www.cellsignal.com/products/primary-antibodies/a-smooth-muscle-actin-d4k9n-xp-rabbit-mab/19245?site-search-type=Products&N=4294956287&Ntt=%C3%8E%C2%B1-smooth+muscle+actin+antibody&fromPage=plp	Cell Signaling Technology	1:1000
Vimentin antibody	https://www.cellsignal.com/products/primary-antibodies/vimentin-d21h3-xp-rabbit-mab/5741?site-search-type=Products&N=4294956287&Ntt=vimentin+antibody&fromPage=plp	Cell Signaling Technology	1:2000
GAPDH antibody	http://www.yfxbio.com/product_info.asp?id=3413	YI FEI XUE Biotechnology	1:1000
CD31	https://www.cellsignal.com/products/primary-antibodies/cd31-pecam-1-89c2-mouse-mab/3528?site-search-type=Products&N=4294956287&Ntt=CD31&fromPage=plp	Cell Signaling Technology	1:1000
FSP-1	https://www.cellsignal.com/products/primary-antibodies/s100a4-d9f9d-rabbit-mab/13018?site-search-type=Products&N=4294956287&Ntt=FSP-1&fromPage=plp	Cell Signaling Technology	1:1000
FAP	http://www.affbiotech.com/goods-879-AF0739-FAP-1+Antibody.html	Affinity Biosciences	1:1000
PDGFR-β	https://www.cellsignal.com/products/primary-antibodies/phospho-pdgf-receptor-a-tyr849-pdgf-receptor-b-tyr857-c43e9-rabbit-mab/3170?site-search-type=Products&N=4294956287&Ntt=pdgfr-%CE%B2&fromPage=plp	Cell Signaling Technology	1:1000
Tublin	http://www.beyotime.com/product/AT819.htm	Beyotime	1: 1000
E-Cadherin antibody	https://www.cellsignal.com/products/primary-antibodies/e-cadherin-24e10-rabbit-mab/3195?site-search-type=Products&N=4294956287	Cell Signaling Technology	1:1000
Ac-STAT3^{Lsy685} antibody	https://www.cellsignal.com/products/primary-antibodies/acetyl-stat3-lys685-antibody/2523?site-search-type=Products&N=4294956287&Ntt=stat3&fromPage=plp	Cell Signaling Technology	1:1000
p-STAT3^{Tyr705} antibody	https://www.cellsignal.com/products/primary-antibodies/phospho-stat3-tyr705-d3a7-xp-rabbit-mab/9145?site-search-type=Products&N=4294956287&Ntt=stat3&fromPage=plp	Cell Signaling Technology	1:1000
STAT3 antibody	https://www.cellsignal.com/products/primary-antibodies/stat3-124h6-mouse-mab/9139?site-search-type=Products&N=4294956287&Ntt=stat3&fromPage=plp	Cell Signaling Technology	1:1000
β-Actin antibody	http://www.beyotime.com/product/AA128.htm	Beyotime	1:1000

Table S2 Primers used in this study

Names	Primers
IL6	F: 5′-AGTAGTGAGGAACAAGCCAGA-3′<br>R: 5′-TACATTTGCCGAAGAGCC-3′
FGF2	F: 5′-AGAAGAGCGACCCTCACATCA-3′<br>R: 5′-CGGTTAGCACACACTCCTTTG-3′
TGF-β	F: 5′-GGCCAGATCCTGTCCAAGC-3′<br>R: 5′-GTGGGTTTCCACCATTAGCAC-3′
MMP2	F: 5′-GCTGGGAGCATGGCGATGGATACC-3′<br>R: 5′-GGACAGAAGCCGTACTTGCCATCC-3′
MMP9	F: 5′-GATGCGTGGAGAGTCGAAAT-3′<br>R: 5′-CACCAAACTGGATGACGATG-3′
VE-cadherin	F: 5′-GTTCACCTTCTGCGAGGATATG-3′<br>R: 5′-GATGGTGAGGATGCAGAGTAAG-3′
α-Smooth muscle actin	F: 5′-CTATGAGGGCTATGCCTTGCC-3′<br>R: 5′-GCTCAGCCAGTAGTAACGAAGGA-3′
Vimentin	F: 5′-TGCCGTTGAAGCTGCTAACTA-3′<br>R: 5′-CCAGAGGGAGTGAATCCAGATTA-3′
β-Actin	F: 5′-TCAGGTCATCACTATCGGCAAT-3′<br>R: 5′-AAAGAAAGGGTGTAAAACGCA-3′

Table S3 siRNAs used in this study

Names	Web Link	Source	Used
STAT3 siRNA	https://datasheets.scbt.com/sc-29493.pdf	Santa Cruz Biotechnology	25 nM
NC siRNA	http://datasheets.scbt.com/sc-37007.pdf	Santa Cruz Biotechnology	25 nM

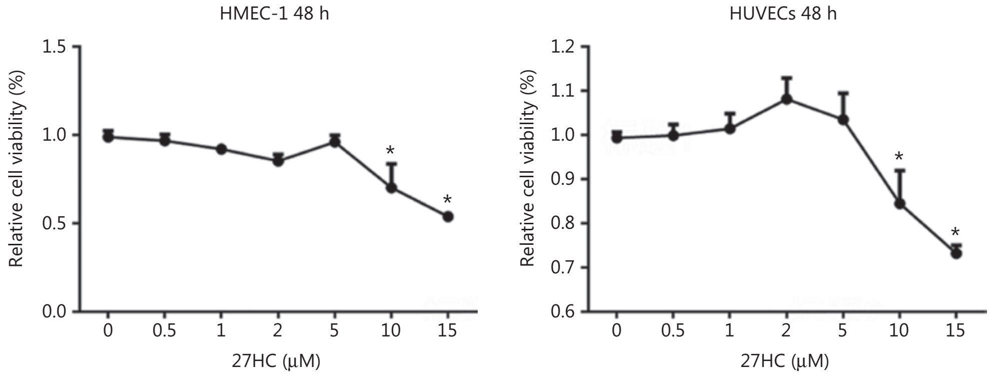

Figure S1 Viability of endothelial cells (ECs) treated with 0.0–5.0 μM 27-hydroxycholesterol (27HC) for 48 h. HMEC-1 and human umbilical vein endothelial cells (HUVECs) (1×10^6 cells) were cultured in 96-well plates for 24 h, and then treated with 0, 0.5, 1, 2, 5, 10, or 15 μM 27-hydroxycholesterol (27HC) for 48 h. Next the culture solution containing 27HC was replaced with 100 μL of cell culture medium containing 10% Cell Counting Kit-8 reagent (CCK-8; Beyotime Co. Ltd) for 1 h. The absorbance at 450 nm was examined by a multi-well plate reader (Model 680, Bio-Rad).

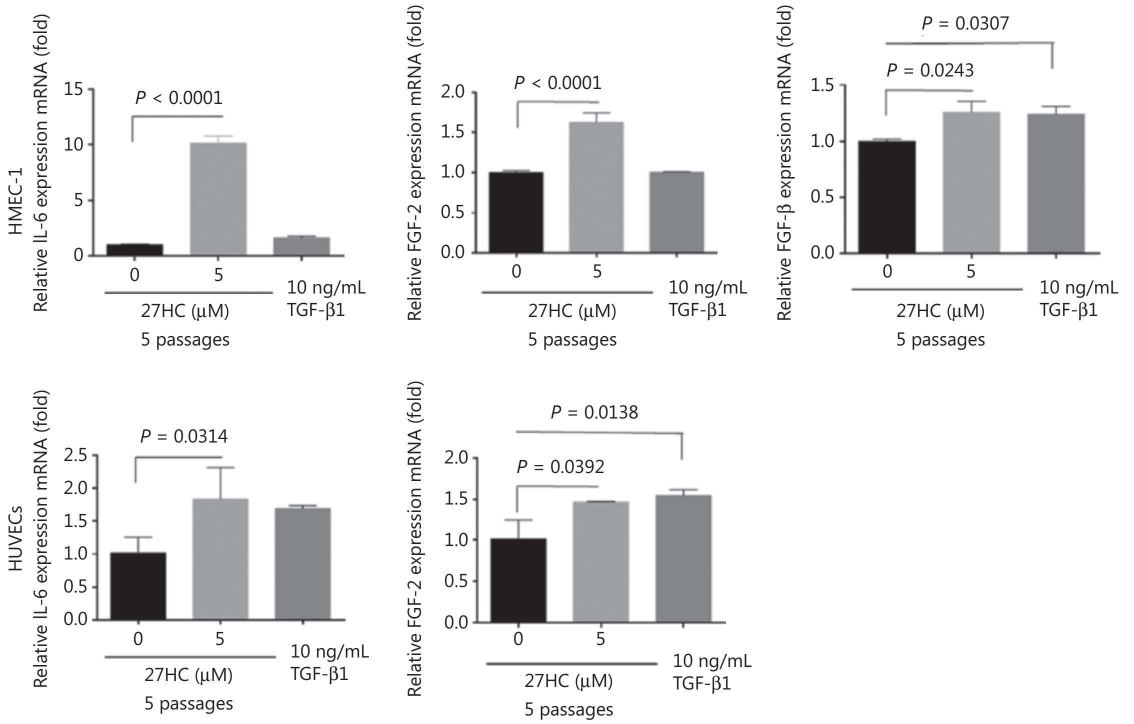

Figure S2 Quantitative real time polymerase chain reaction (qRT-PCR) analyses of mRNA expression in cancer-associated fibroblast (CAF) secretions in endothelial cells (ECs) treated with 27-hydroxycholesterol (27HC). HMEC-1 cells were exposed to 0 or 5 μM 27HC for 5 passages. In triplicate of interleukin (IL)-6, fibroblast growth factor (FGF)-2 and transforming growth factor (TGF)-β mRNAs were determined by qRT-PCR. TGF-β1 (10 ng/mL) treatment was used as a positive control. Human umbilical vein endothelial cells (HUVECs) were treated with 0, or 5 μM of 27HC for 5 passages, and in triplicate of IL-6 and FGF-2 mRNAs were determined by qRT-PCR. TGF-β1 (10 ng/mL) was used as a positive control.

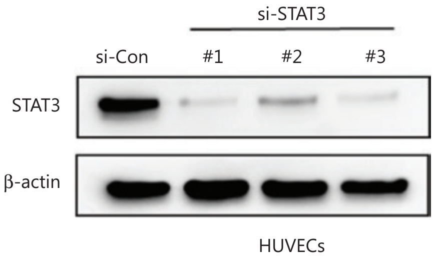

Figure S3 The effects of three si-RNAs specific to STAT3 in human umbilical vein endothelial cells (HUVECs). HUVECs were transiently transfected with si-Con or 3 different si-STAT3 for 6 h. Western blot analyses of the protein expressions of STAT3.

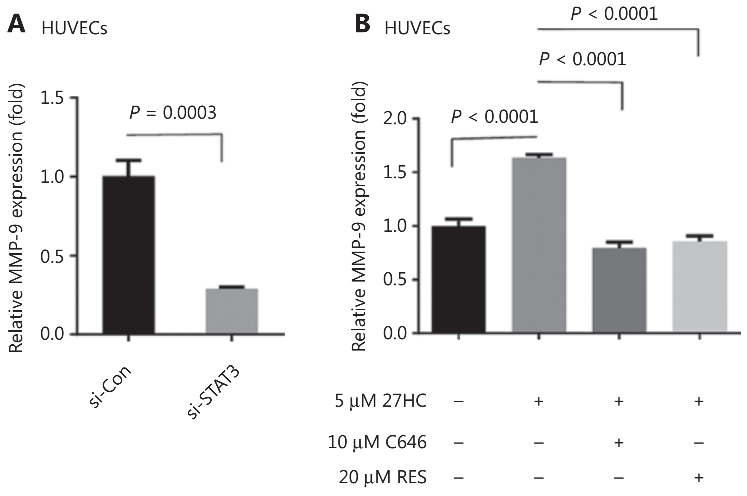

Figure S4 The effect of STAT3 on MMP9 in human umbilical vein endothelial cells (HUVECs). (A) Human umbilical vein endothelial cells (HUVECs) were transiently transfected with si-Con or si-STAT3 for 6 h. MMP9 mRNA expression was determined by quantitative real time polymerase chain reaction (qRT-PCR). (B) HUVECs were pre-treated with C646 (0, 10 μM) and RES (0, 20 μM) for 3 h, followed by treatment with 0 or 5 μM 27-hydroxycholesterol (27HC) for another 48 h. Then MMP9 mRNA expression was determined by qRT-PCR.
